# Genomic Encyclopedia of Type Strains, Phase I: The one thousand microbial genomes (KMG-I) project

**DOI:** 10.4056/sigs.5068949

**Published:** 2013-12-17

**Authors:** Nikos C. Kyrpides, Tanja Woyke, Jonathan A. Eisen, George Garrity, Timothy G. Lilburn, Brian J. Beck, William B. Whitman, Phil Hugenholtz, Hans-Peter Klenk

**Affiliations:** 1 DOE Joint Genome Institute, Walnut Creek, CA; 2 University of California, Davis, CA; 3 Department of Microbiology and Molecular Genetics, Michigan State University, East Lansing, Michigan, USA; 4 NamesforLife, LLC, East Lansing, MI, USA; 5 American Type Culture Collection, Manassas, VA; 6 Department of Microbiology, University of Georgia, Athens, GA; 7 Australian Centre for Ecogenomics, The University of Queensland, Brisbane QLD 4072, Australia; 8 Leibniz Institute DSMZ — German Collection of Microorganisms and Cell Cultures, Braunschweig, Germany

## Abstract

The Genomic Encyclopedia of Bacteria and Archaea (GEBA) project was launched by the JGI in 2007 as a pilot project with the objective of sequencing 250 bacterial and archaeal genomes. The two major goals of that project were (a) to test the hypothesis that there are many benefits to the use the phylogenetic diversity of organisms in the tree of life as a primary criterion for generating their genome sequence and (b) to develop the necessary framework, technology and organization for large-scale sequencing of microbial isolate genomes. While the GEBA pilot project has not yet been entirely completed, both of the original goals have already been successfully accomplished, leading the way for the next phase of the project.

Here we propose taking the GEBA project to the next level, by generating high quality draft genomes for 1,000 bacterial and archaeal strains. This represents a combined 16-fold increase in both scale and speed as compared to the GEBA pilot project (250 isolate genomes in 4+ years). We will follow a similar approach for organism selection and sequencing prioritization as was done for the GEBA pilot project (i.e. phylogenetic novelty, availability and growth of cultures of type strains and DNA extraction capability), focusing on type strains as this ensures reproducibility of our results and provides the strongest linkage between genome sequences and other knowledge about each strain. In turn, this project will constitute a pilot phase of a larger effort that will target the genome sequences of all available type strains of the *Bacteria* and *Archaea*.

## Introduction

### I. The importance of the research

In June 2011, there were approximately 9,000 bacterial and archaeal species with validly published names, and with rare exceptions there was a viable sample of the designated type strain available from one or more service culture collections. Although commonly misconstrued as the archetypal representative of a species, type strains are instead live specimens that serve as a fixed reference points to which bacterial and archaeal names are attached. These names (and by reference the type strains) provide an entry into the literature, databases, and our knowledge of microbial diversity. Thus, type strains play a crucial role in defining the phylogenomic and taxonomic space of *Bacteria* and *Archaea*. By definition, type strains are descendants of the original isolates that were defined in species and subspecies descriptions, as requested by the Bacteriological Code [1] and exhibit all of the relevant phenotypic and genotypic properties cited in the original published taxonomic circumscriptions. Except in cases such as some symbionts and other non-cultivable species defined prior to the 1999 revisions to the rules [2-4], virtually all type strains are available in pure axenic cultures.

**Figure 1:**
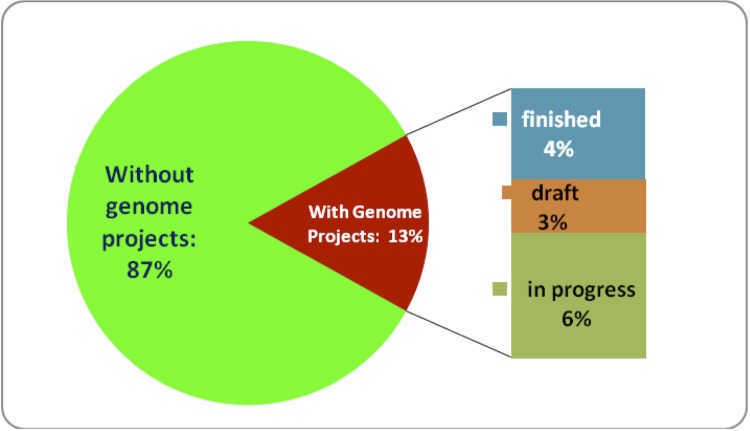
Genome project coverage of bacterial and archaeal type strains as of June 2011. From a total of approximately 9,000 bacterial and archaeal type strains, 1219 (13%) (non-redundant) have a publicly known genome project.

Although the Genomic Encyclopedia of Bacteria and Archaea (GEBA) pilot project [5] provided a significant boost in the number of sequenced type strains, upon its completion only 13% of the total number of available type strains were sequenced [6]. From that fraction, only 7% had sequence data available as complete or draft sequences (Figure 1). Given that the number of newly described species and subspecies (along with the relevant type strains) increases by about 600 per year, the overall coverage of taxonomic types by genome sequencing projects has remained stable during the last several years even as more sequences have been completed.

In addition, the number of genome projects for the currently defined type strains represent about 13% of the overall number of registered genome sequencing projects for bacterial and archaeal strains [6].

Genomic sequencing of all of the type strains is an important goal. This proposal represents the first phase of this effort, setting the goal of sequencing the first 1,000 of those remaining type strains. In the GEBA pilot project [5] we asked: *How much of the known phylogenetic diversity of Bacteria and Archaea has been sampled?* Here in KMG1, we extend that question to: *How quickly, thoroughly and efficiently can we fill in the gaps and extend our knowledge of the cultivated Bacterial and Archaeal species?*

Much of the scientific value and anticipated advantages of such a large effort [7,8] has already been provided from the much smaller scale of the GEBA pilot project [5]. We have addressed this by estimating the diversity of all the cultured species of *Bacteria* and *Archaea* (based on the availability of their 16S rRNA sequences) and quantified how much of that diversity has already been sampled.

**Figure 2:**
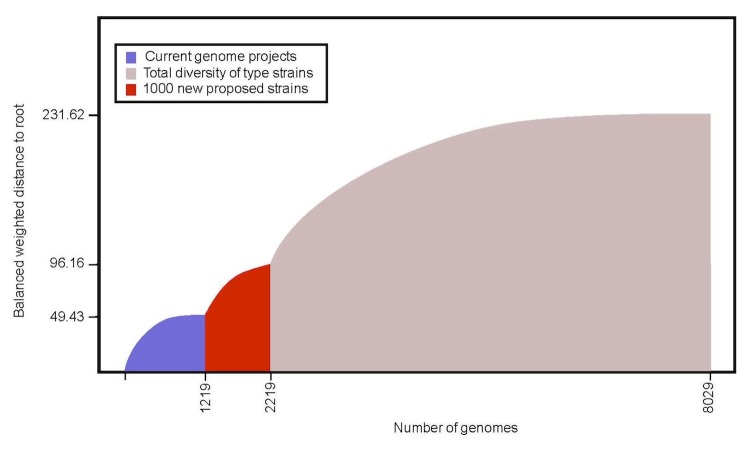
Phylogenetic diversity of the type strains of *Bacteria* and *Archaea* based on the SSU rRNA genes as of June 2011. Blue: phylogenetic diversity of current complete and ongoing genome projects from 1,219 type strains (GOLD 1/2011); Red: of the 1,000 type strains proposed to be sequenced here; Pink: phylogenetic diversity of all type strains available at the Living Tree Project (LTP) [9]. All our calculations are based on the LTP tree from September 2010 (latest version) * which contains 8,029 of the about 9,000 type strains.

As shown on Figure 2, with the list of the 1,000 type strains we propose to sequence here, we aim to cover over 40% of the phylogenetic diversity of cultured members of the *Bacteria* and *Archaea* (quantified as the overall distance on the 16S rRNA tree). In fact with this proposed list, we will nearly double the currently available phylogenetic diversity of the type strains with finished and ongoing genome sequencing projects.

It should be noted here that while the current list of the type strains with completed and ongoing genome projects (blue color on Fig. 2) is about 1,200, there has not been a systematic effort to sequence type strains, and these represent on a small fraction of the over 7,700 genome projects completed in well over 15 years of microbial genome sequencing. Since a list of 1,000 strains would be too long to list here, we have attached the complete list on a separate file, and we present here only a summary table of the number of organisms we intend to cover per phylum (Table 1).

**Table 1:** Summary table for KMG project (including non-redundant non-type strains)

**Phylum**	**Type strains**	**Species** **/ subsp.**	**%** **Synonyms**	**Type** **Genome sequences**	**Non-type Genome Sequences**	**Coverage**	**Type** **Genomes** **proposed**
*Crenarchaeota*	57	61	6.6	33	0	57.9	**-**
*Euryarchaeota*	314	388	19.1	143	4	45.5	**9**
*Thaumarchaeota*	1	1	0.0	1	0	100.0	**-**
*Aquificae*	29	31	6.5	8	0	27.6	**2**
*Thermotogae*	37	38	2.6	14	1	37.8	**13**
*Thermodesulfobacteria*	7	8	12.5	2	1	28.6	**4**
*Deinococcus-Thermus*	71	76	6.6	19	0	26.8	**18**
*Chrysiogenetes*	4	4	0.0	2	0	50.0	**-**
*Chloroflexi*	27	28	3.6	11	0	40.7	**-**
*Nitrospirae*	12	12	0.0	2	0	16.7	**3**
*Deferribacteres*	12	12	0.0	6	0	50.0	**1**
*Cyanobacteria*	88	90	2.2	9	2	10.2	**-**
*Chlorobi*	16	22	27.3	9	0	56.3	**-**
*Proteobacteria*	3,541	4,323	18.1	364	35	10.3	**385**
*Firmicutes*	1,875	2,263	17.1	311	14	16.6	**285**
*Tenericutes*	234	258	9.3	25	0	10.7	**2**
*Actinobacteria*	2,439	2,953	17.4	145	5	5.9	**129**
*Planctomycetes*	15	19	21.1	10	0	66.7	**2**
*Chlamydiae*	17	20	15.0	8	0	47.1	**-**
*Spirochaetes*	112	127	11.8	25	0	22.3	**2**
*Fibrobacteres*	3	5	40.0	1	0	33.3	**-**
*Acidobacteria*	11	11	0.0	3	0	27.3	**-**
*Bacteroidetes*	767	914	16.1	131	9	17.1	**134**
*Fusobacteria*	38	47	19.1	12	0	31.6	**7**
*Verrucomicrobia*	35	35	0.0	6	2	17.1	**3**
*Gemmatimonadetes*	1	1	0.0	1	0	100.0	**-**
*Dictyoglomi*	2	3	33.3	2	0	100.0	**-**
*Lentisphaerae*	2	2	0.0	1	0	50.0	**-**
*Synergistetes*	17	18	5.6	10	1	58.8	**1**
*Caldiserica*	1	1	0.0	1	0	100.0	**-**
*Elusimicrobia*	1	1	0.0	1	0	100.0	**-**
**Total**	**9,786**	**11,772**	**16.8**	**1,316**	**74**	**14.2**	**1,000**

Totals are based on an export of the *Bacterial* and *Archaeal* taxonomic and nomenclatural events in the NamesforLife Database on May 29, 2011. There are 33 named phyla that are currently in common usage to which the validly named species and subspecies were mapped. Percent synonyms were determined based on the number of recorded assertions of homotypic synonymy, new combinations and coordinate status species and subspecies. Heterotypic synonyms are not included in this number as they have separate type strains. Genome sequences are based on those that are declared as types from the GOLD (5/28/2011). *Cyanobacteria* species are based on those species described in *Bergey’s Manual of Systematic Bacteriology*, Vol 1., 2^nd^ Ed. 2001 and represent the dominant morphotypes. Non-redundant non-type strains, bearing validly published names, for which types have yet to be sequenced are added to the table to minimize potential for overlap.

### II. Project design

**(a) Selection of target organisms:** Target organisms were selected based on a score that measures the relative contribution of each species to the total phylogenetic diversity (PD), as inferred from a phylogenetic tree with computed branch lengths [10]. The underlying phylogenetic tree was the one from the All-Species-Living-Tree-Project in the latest available version from 9/2010, comprising 8,029 leaves (species/subspecies). Species with ongoing or completed genome sequencing projects registered in GOLD (1/2011) were excluded and the highest scoring (PD) species were screened for growth conditions that allow the production of sufficient cell past for DNA extraction. One thousand prime targets were selected and augmented by an additional 685 to serve as a back-up in event of failure to produce sufficient DNA or the inevitability that another research group may have begun sequencing the same organism.

**(b) Organism growth and nucleic acid isolation**: All of the proposed strains will be grown at the DSMZ [http://www.dsmz.de/] and ATCC [http://www.atcc.org/] culture collection centers. Both bioresource centers have extensive experience in growing microbial organisms under a wide range of conditions including extremely high temperatures or salt concentration. The new sequencing technologies that will be employed for this project, coupled with an understanding that completely sequenced genomes are not necessarily required to achieve our goals, means that significantly less DNA is needed per individual organism. The current sequencing protocol requires as little as 10 micrograms of DNA for high quality draft genome sequencing, which will greatly facilitate the progress of this project. RNA isolation under a single steady state condition will also be performed at DSMZ.

**(c) Sequencing, data QC and assembly**: The combination of high throughput sequencing based on the HiSeq Illumina technology, together with our ability to multiply individual channels per flowcell, will allow us to complete this project at a low per unit cost (<$100 per genome, unburdened cost). The current plan will be to pool 48-96 microbes per HiSeq channel assuming no major changes in sequencing throughput. This will provide higher than 100× coverage per microbial genome, and will bring down the total sequencing allocation requested for the 1,000 genomes to a total of 1.5-3 runs. Continued improvements and automation of the JGI sequence QC pipeline (“rolling QC”) will enable a largely *hands-off* approach for the quality assessment and assembly of the sequence data. Although we expect that minor manual intervention will be needed at this stage. Concurrent improvements in bioinformatics and computational power will allow us to assemble most targeted genomes into well under 100 major contigs.

**(d) Annotation and comparative analysis**: The microbial genome annotation pipeline at the JGI has been scaled to handle hundreds of microbial genomes per month [9,11,12].

**(e) Publication of analyzed genomes**: Although not all of the draft genomes we propose to generate will be of publication quality, we still plan to publish as many as possible in *Standards in Genomic Sciences* [13,14]. We will use a software pipeline from the GEBA pilot project for accelerated generation of standardized manuscript text that allows (i) automatized collection of sequences and metadata for incorporation in tables and/or text of the manuscripts, (ii) 16S rRNA sequence comparison with the Greengenes database to analyze the target occurrence of the target strain in the environment, including a statistical analysis of keywords (iii) 16S rRNA-based phylogenetic analysis and generation of publication-quality tree graphics. Data are provided to the authors *via* a HTML-based template system. More code for the automated generation of phylogenetic and functional analyses will be available by the time of the proposed start date of the project.

### III. The scientific questions we expect to answer

The derived data will be utilized in several different ways. We already know from projects such as the GEBA pilot project [5] and the Human Microbiome Project (HMP) project [15] that reference genomes provide significant support in the analysis of metagenomic datasets. It is now well-established that the road to success in metagenomics is through microbial genomics. A systematic coverage of the tree of life will further support several ongoing metagenomic studies. KMG is also strongly endorsed by members of the scientific community leading large international metagenomics studies. The discovery of novel protein families, or novel members of already known protein families, and the overall impact on genome annotation has also been previously documented with much smaller scale sequencing efforts.

In addition to the typical ways the data will be analyzed and compared, and beyond the general evolutionary questions that were posed and explored during the GEBA pilot project, we plan to engage the larger scientific community to work with us and lead various types of additional analyses. Specifically, we plan to organize and facilitate the work of the community either around special phylogenetic groups, around special topics of comparative analysis, and around topics of relevance to the DOE mission (bioenergy, bioremediation, carbon sequestration). This will ensure the maximum benefit of the data derived from this project. This will also be coupled with NamesforLife’s [http://services.namesforlife.com/] ongoing efforts to mine the world’s patent literature and to make important connections between what is known about microbes in nature and what is known about the practical uses of these organisms.

### IV. The size and nature of the larger community that will use the data

We plan to immediately release the data to the public through a variety of channels. Because no restrictions will be associated with the release of the data, the community will be able to make immediate and full use of our findings. The grand scale of the project, the lack of focus on specific applications, and the emphasis on microbial diversity and the tree of life, will most certainly generate novel information of broad scientific interest and benefit for the community at large.

The GEBA pilot project has already generated a great deal of interest in the life sciences research community as well as amongst biology educators, based on the fact that these genomes were released to the community without any restrictions.

The GEBA pilot project has also developed new standards in relation to metadata. The genomes already released to Genbank, have some of the most enriched metadata fields available as compared to any other genome sequencing projects. We will continue with this effort, and major emphasis will be given to the curation and public release of the associated metadata for all of these organisms. This will be done in collaboration and coordination with the Genomic Standards Consortium [16], and we will comply with the recently proposed Minimum Information for Genomics Sequences (MIGS) metadata [14,17].

### V. The relevance of the project to problems of societal importance

The selection of sequencing targets will not be based on their specific relevance with a scientific mission, per-se. However, as the GEBA pilot project has demonstrated, a phylogeny-based selection process for sequencing will generate tremendous amounts of fundamental knowledge and information that will impact many fields and realms of science, medicine and technology. As an example, we have shown that the phylogenetic basis of genome project selection is increasing the probability of novel protein family discovery [5]. This is certainly expected to lead to the discovery of novel enzymatic activities with direct relevance to bioenergy, biogeochemistry, bioremediation and carbon cycling applications. We have already published a number of papers based on GEBA pilot genomes demonstrating this fact and several more are currently submitted or in preparation [18-20]. A notable example of one of these studies involves the identification of a number of novel cellulases from a halophilic archaeon (not previously suspected to be an organism likely to harbor cellulases), which were followed by experimental verification [18, 19].
